# 5-Ethyl-4-phenyl-1*H*-pyrazol-3(2*H*)-one

**DOI:** 10.1107/S1600536811001589

**Published:** 2011-01-15

**Authors:** Wan-Sin Loh, Hoong-Kun Fun, R. Venkat Ragavan, V. Vijayakumar, M. Venkatesh

**Affiliations:** a X-ray Crystallography Unit, School of Physics, Universiti Sains Malaysia, 11800 USM, Penang, Malaysia; b Organic Chemistry Division, School of Advanced Sciences, VIT University, Vellore 632 014, India

## Abstract

The asymmetric unit of the title compound, C_11_H_12_N_2_O, consists of two crystallographically independent mol­ecules (*A* and *B*) with similar geometries. Both mol­ecules exist in a keto form, the C=O bond length being 1.286 (2) Å in *A* and 1.283 (2) Å in *B*. The dihedral angles between the pyrazole ring and the attached phenyl ring are 43.28 (12) and 46.88 (11)°, respectively, for *A* and *B*. The ethyl unit in mol­ecule *B* is disordered over two positions with a site-occupancy ratio of 0.508 (5):0.492 (5). In the crystal, each of the independent mol­ecules forms a centrosymmetric dimer with an *R*_2_^2^(8) ring motif through a pair of N—H⋯O hydrogen bonds. These dimers are further connected into a three-dimensional network by inter­molecular N—H⋯O and C—H⋯O hydrogen bonds. Inter­molecular C—H⋯π inter­actions are also present.

## Related literature

For background to pyrazole derivatives and their microbial activity, see: Ragavan *et al.* (2009 ▶, 2010 ▶). For bond-length data, see: Allen *et al.* (1987 ▶). For related structures, see: Loh *et al.* (2010 ▶, 2010*a* ▶,*b* ▶, 2011 ▶). For hydrogen-bond motifs, see: Bernstein *et al.* (1995 ▶). For the stability of the temperature controller used in the data collection, see: Cosier & Glazer (1986 ▶).
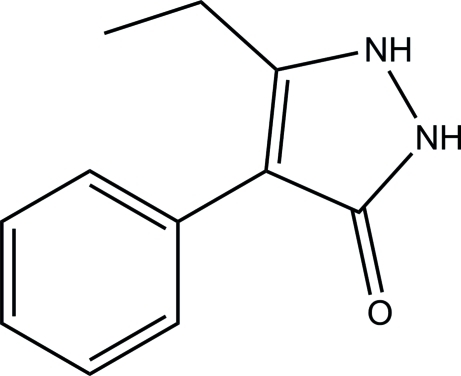


## Experimental

### 

#### Crystal data

C_11_H_12_N_2_O
*M* *_r_* = 188.23
Monoclinic, 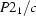

*a* = 11.0898 (3) Å
*b* = 13.2171 (4) Å
*c* = 15.0265 (5) Å
β = 114.539 (2)°
*V* = 2003.58 (11) Å^3^
*Z* = 8
Mo *K*α radiation
μ = 0.08 mm^−1^
*T* = 100 K
0.60 × 0.16 × 0.13 mm

#### Data collection

Bruker SMART APEXII CCD area-detector diffractometer
Absorption correction: multi-scan (*SADABS*; Bruker, 2009 ▶) *T*_min_ = 0.953, *T*_max_ = 0.989
22130 measured reflections
5845 independent reflections
3654 reflections with *I* > 2σ(*I*)
*R*_int_ = 0.063

#### Refinement

*R*[*F* ^2^ > 2σ(*F* ^2^)] = 0.065
*wR*(*F* ^2^) = 0.166
*S* = 1.05
5845 reflections
284 parameters
2 restraints
H atoms treated by a mixture of independent and constrained refinement
Δρ_max_ = 0.38 e Å^−3^
Δρ_min_ = −0.30 e Å^−3^

### 

Data collection: *APEX2* (Bruker, 2009 ▶); cell refinement: *SAINT* (Bruker, 2009 ▶); data reduction: *SAINT*; program(s) used to solve structure: *SHELXTL* (Sheldrick, 2008 ▶); program(s) used to refine structure: *SHELXTL*; molecular graphics: *SHELXTL*; software used to prepare material for publication: *SHELXTL* and *PLATON* (Spek, 2009 ▶).

## Supplementary Material

Crystal structure: contains datablocks global, I. DOI: 10.1107/S1600536811001589/is2655sup1.cif

Structure factors: contains datablocks I. DOI: 10.1107/S1600536811001589/is2655Isup2.hkl

Additional supplementary materials:  crystallographic information; 3D view; checkCIF report

## Figures and Tables

**Table 1:** Hydrogen-bond geometry (Å, °) *Cg*1 and *Cg*2 are the centroids of the C4*B*–C9*B* and C4*A*–C9*A* rings, respectively.

*D*—H⋯*A*	*D*—H	H⋯*A*	*D*⋯*A*	*D*—H⋯*A*
N1*B*—H1*NB*⋯O1*A*	1.00 (2)	1.73 (2)	2.700 (2)	161 (2)
N2*B*—H2*NB*⋯O1*B*^i^	1.02 (2)	1.72 (2)	2.738 (2)	176 (2)
N2*A*—H2*NA*⋯O1*A*^ii^	0.98 (3)	1.74 (3)	2.704 (2)	171 (2)
N1*A*—H1*NA*⋯O1*B*^iii^	0.98 (3)	1.74 (3)	2.691 (2)	162 (2)
C8*A*—H8*AA*⋯O1*A*^iv^	0.93	2.47	3.370 (3)	163
C10*A*—H10*C*⋯*Cg*1^iii^	0.97	2.61	3.464 (2)	147
C10*B*—H10*E*⋯*Cg*2	0.97	2.71	3.524 (3)	142

Symmetry codes: (i) 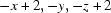; (ii) 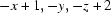; (iii) 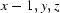; (iv) 
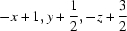
.

**Fig. 1.**
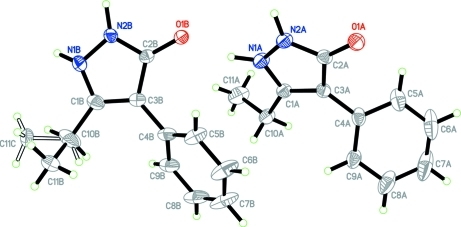
The molecular structure of the title compound, showing 50% probability displacement ellipsoids and the atom-numbering scheme. Open bonds indicate the minor component.

**Fig. 2.**
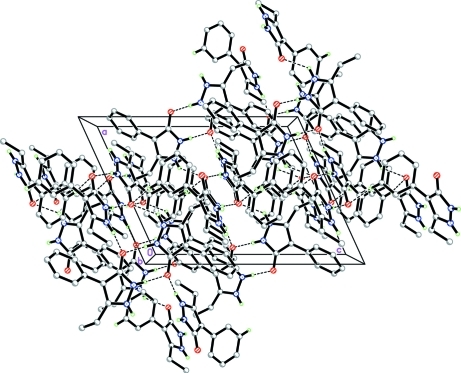
The crystal packing of the title compound, showing the three-dimensional network. Only the major component is shown. H atoms not involved in the intermolecular interactions (dashed lines) have been omitted for clarity.

## supplementary crystallographic information

### Comment

Antibacterial and antifungal activities of the azoles are most widely studied and some of them are in clinical practice as anti-microbial agents. However, the azole-resistant strains had led to the development of new anti-microbial compounds. In particular, pyrazole derivatives are extensively studied and used as anti-microbial agents. Pyrazole is an important class of heterocyclic compounds and many pyrazole derivatives are reported to have the broad spectrum of biological properties such as anti-inflammatory, antifungal, herbicidal, anti-tumour, cytotoxic, molecular modelling and antiviral activities. Pyrazole derivatives also act as anti-angiogenic agents, A3 adenosine receptor antagonists, neuropeptide YY5 receptor antagonists as well as kinase inhibitor for treatment of type 2 diabetes, hyperlipidemia, obesity and thrombopiotinmimetics. Recently urea derivatives of pyrazoles have been reported as potent inhibitors of p38 kinase. Since the high electronegativity of halogens (particularly chlorine and fluorine) in the aromatic part of the drug molecules play an important role in enhancing their biological activity, we are interested to have 4-fluoro or 4-chloro substitution in the aryls of 1,5-diaryl pyrazoles. As part of our on-going research aiming the synthesis of new anti-microbial compounds, we have reported the synthesis of novel pyrazole derivatives and their microbial activities (Ragavan *et al.*, 2009, 2010).

The title compound (Fig. 1), consists of two crystallographically independent molecules, with similar geometries and exist in keto-form with the bond length of C═O being 1.286 (2) Å in molecule *A* and 1.283 (2) Å in molecule *B*. This indicates that the compound undergoes an enol-to-keto tautomerism during the crystallization process In molecule *A*, the pyrazole ring (N1A/N2A/C1A–C3A) is approximately planar [maximum deviation of 0.0262 (16) Å at N2A] and forms a dihedral angle of 43.28 (12)° with the attached phenyl ring (C4A–C9A). In molecule *B*, the pyrazole ring (N1B/N2B/C1B–C3B) is approximately planar with a maximum deviation of 0.0209 (15) Å at N1B and form a dihedral angle of 46.88 (11)° with the attached phenyl ring (C4B–C9B). The ethyl unit (C10B/C11B) in the molecule *B* is observed to be disordered over two positions with a site-occupancy ratio of 0.508 (5):0.492 (5). Bond lengths (Allen *et al.*, 1987) and angles are within the normal ranges and are comparable to the related structures (Loh *et al.*,  2010, 2011; Loh *et al.*,  2010*a*,*b*).

In the crystal packing (Fig. 2), intermolecular N2A—H2NA···O1A and N2B—H2NB···O1B hydrogen bonds (Table 1) link the neighbouring molecules to form dimers, generating *R*_2_^2^(8) ring motifs (Bernstein *et al.*, 1995) and are further packed into three-dimensional network by intermolecular N1B—H1NB···O1A, N1A—H1NA···O1B and C8A—H8AA···O1A hydrogen bonds (Table 1). The crystal structure is further stabilized by C—H···π interactions (Table 1) involving *Cg*1 (C4B–C9B) and *Cg*2 (C4A–C9A).

### Experimental

The compound has been synthesized using the method available in the literature (Ragavan *et al.*, 2010) and recrystallized using the ethanol-chloroform 1:1 mixture (yield 81%, *m. p.* 361.3–362.1 K).

### Refinement

N-bound H atoms were located from a difference Fourier map and were refined freely [N—H = 0.97 (2) to 1.02 (2) Å]. The remaining H atoms were positioned geometrically with the bond length of C—H = 0.93 to 0.97 Å and were refined using a riding model, with *U*_iso_(H) = 1.2 or 1.5*U*_eq_(C). A rotating group model was applied to the methyl groups. The ethyl unit of molecule *B* was disordered over two positions with a site-occupancy of 0.508 (5):0.492 (5). Bond-distance restraints were applied for C10B—C11B and C10B—C11C.

### Crystal data

**Table:**

C_11_H_12_N_2_O	*F*(000) = 800
*M**_r_* = 188.23	*D*_x_ = 1.248 Mg m^−^^3^
Monoclinic, *P*2_1_/*c*	Mo *K*α radiation, λ = 0.71073 Å
Hall symbol: -P 2ybc	Cell parameters from 4628 reflections
*a* = 11.0898 (3) Å	θ = 2.5–30.0°
*b* = 13.2171 (4) Å	µ = 0.08 mm^−^^1^
*c* = 15.0265 (5) Å	*T* = 100 K
β = 114.539 (2)°	Needle, colourless
*V* = 2003.58 (11) Å^3^	0.60 × 0.16 × 0.13 mm
*Z* = 8	

### Data collection

**Table:**

Bruker SMART APEXII CCD area-detector diffractometer	5845 independent reflections
Radiation source: fine-focus sealed tube	3654 reflections with *I* > 2σ(*I*)
graphite	*R*_int_ = 0.063
φ and ω scans	θ_max_ = 30.1°, θ_min_ = 2.1°
Absorption correction: multi-scan (*SADABS*; Bruker, 2009)	*h* = −15→15
*T*_min_ = 0.953, *T*_max_ = 0.989	*k* = −18→18
22130 measured reflections	*l* = −20→21

### Refinement

**Table:**

Refinement on *F*^2^	Secondary atom site location: difference Fourier map
Least-squares matrix: full	Hydrogen site location: inferred from neighbouring sites
*R*[*F*^2^ > 2σ(*F*^2^)] = 0.065	H atoms treated by a mixture of independent and constrained refinement
*wR*(*F*^2^) = 0.166	*w* = 1/[σ^2^(*F*_o_^2^) + (0.0659*P*)^2^ + 0.5295*P*] where *P* = (*F*_o_^2^ + 2*F*_c_^2^)/3
*S* = 1.05	(Δ/σ)_max_ = 0.001
5845 reflections	Δρ_max_ = 0.38 e Å^−^^3^
284 parameters	Δρ_min_ = −0.30 e Å^−^^3^
2 restraints	Extinction correction: *SHELXTL* (Sheldrick, 2008), Fc^*^=kFc[1+0.001xFc^2^λ^3^/sin(2θ)]^-1/4^
Primary atom site location: structure-invariant direct methods	Extinction coefficient: 0.0163 (19)

### Special details

**Table:**

Experimental. The crystal was placed in the cold stream of an Oxford Cryosystems Cobra open-flow nitrogen cryostat (Cosier & Glazer, 1986) operating at 100.0 (1) K.
Geometry. All e.s.d.'s (except the e.s.d. in the dihedral angle between two l.s. planes) are estimated using the full covariance matrix. The cell e.s.d.'s are taken into account individually in the estimation of e.s.d.'s in distances, angles and torsion angles; correlations between e.s.d.'s in cell parameters are only used when they are defined by crystal symmetry. An approximate (isotropic) treatment of cell e.s.d.'s is used for estimating e.s.d.'s involving l.s. planes.
Refinement. Refinement of *F*^2^ against ALL reflections. The weighted *R*-factor *wR* and goodness of fit *S* are based on *F*^2^, conventional *R*-factors *R* are based on *F*, with *F* set to zero for negative *F*^2^. The threshold expression of *F*^2^ > σ(*F*^2^) is used only for calculating *R*-factors(gt) *etc*. and is not relevant to the choice of reflections for refinement. *R*-factors based on *F*^2^ are statistically about twice as large as those based on *F*, and *R*- factors based on ALL data will be even larger.

### Fractional atomic coordinates and isotropic or equivalent isotropic displacement parameters (Å^2^)

**Table:**

	*x*	*y*	*z*	*U*_iso_*/*U*_eq_	Occ. (<1)
O1A	0.59450 (13)	0.07629 (9)	0.94578 (11)	0.0314 (3)	
N1A	0.29084 (17)	0.16378 (11)	0.92423 (13)	0.0288 (4)	
N2A	0.38633 (15)	0.09048 (11)	0.94491 (12)	0.0271 (4)	
C1A	0.32664 (18)	0.24453 (13)	0.88567 (14)	0.0246 (4)	
C2A	0.48806 (18)	0.12736 (13)	0.92718 (14)	0.0244 (4)	
C3A	0.44982 (17)	0.22608 (12)	0.88657 (14)	0.0239 (4)	
C4A	0.53044 (18)	0.29233 (13)	0.85342 (16)	0.0298 (4)	
C5A	0.6668 (2)	0.30005 (15)	0.90864 (18)	0.0396 (5)	
H5AA	0.7066	0.2645	0.9671	0.047*	
C6A	0.7438 (2)	0.36080 (18)	0.8767 (3)	0.0602 (8)	
H6AA	0.8348	0.3652	0.9137	0.072*	
C7A	0.6861 (3)	0.41400 (18)	0.7910 (3)	0.0685 (10)	
H7AA	0.7379	0.4549	0.7704	0.082*	
C8A	0.5513 (3)	0.40705 (19)	0.7351 (2)	0.0604 (8)	
H8AA	0.5124	0.4430	0.6768	0.072*	
C9A	0.4736 (2)	0.34624 (16)	0.76586 (18)	0.0401 (5)	
H9AA	0.3829	0.3415	0.7278	0.048*	
C10A	0.23852 (19)	0.33569 (14)	0.85216 (16)	0.0306 (4)	
H10C	0.1769	0.3258	0.7845	0.037*	
H10D	0.2927	0.3942	0.8546	0.037*	
C11A	0.1600 (2)	0.35798 (17)	0.91215 (19)	0.0450 (6)	
H11D	0.1092	0.4187	0.8884	0.068*	
H11E	0.2199	0.3668	0.9795	0.068*	
H11F	0.1013	0.3025	0.9064	0.068*	
O1B	1.07863 (12)	0.06616 (9)	0.92558 (9)	0.0254 (3)	
N1B	0.73764 (15)	0.06391 (11)	0.83821 (12)	0.0275 (4)	
N2B	0.86312 (14)	0.04445 (11)	0.90754 (12)	0.0228 (3)	
C1B	0.74826 (19)	0.11417 (14)	0.76342 (15)	0.0303 (4)	
C2B	0.95360 (17)	0.07674 (12)	0.87492 (13)	0.0215 (4)	
C3B	0.88097 (18)	0.12138 (13)	0.78120 (14)	0.0251 (4)	
C4B	0.9388 (2)	0.16679 (13)	0.71837 (14)	0.0291 (4)	
C5B	1.0386 (2)	0.11721 (15)	0.70194 (16)	0.0361 (5)	
H5BA	1.0694	0.0549	0.7315	0.043*	
C6B	1.0928 (3)	0.15955 (16)	0.64204 (18)	0.0505 (7)	
H6BA	1.1588	0.1253	0.6312	0.061*	
C7B	1.0483 (3)	0.25308 (17)	0.59830 (17)	0.0541 (7)	
H7BA	1.0840	0.2814	0.5579	0.065*	
C8B	0.9506 (3)	0.30381 (16)	0.61513 (16)	0.0459 (6)	
H8BA	0.9209	0.3665	0.5862	0.055*	
C9B	0.8973 (2)	0.26163 (15)	0.67475 (15)	0.0360 (5)	
H9BA	0.8326	0.2969	0.6862	0.043*	
C10B	0.6280 (2)	0.14952 (18)	0.67717 (18)	0.0499 (6)	
H10A	0.5568	0.1581	0.6980	0.060*	0.508 (5)
H10B	0.6473	0.2157	0.6583	0.060*	0.508 (5)
H10E	0.6172	0.2209	0.6868	0.060*	0.492 (5)
H10F	0.6477	0.1442	0.6202	0.060*	0.492 (5)
C11B	0.5811 (4)	0.0874 (3)	0.5935 (3)	0.0427 (13)	0.508 (5)
H11A	0.5027	0.1168	0.5442	0.064*	0.508 (5)
H11B	0.5608	0.0215	0.6104	0.064*	0.508 (5)
H11C	0.6481	0.0816	0.5689	0.064*	0.508 (5)
C11C	0.5046 (3)	0.1031 (3)	0.6533 (4)	0.0395 (13)	0.492 (5)
H11G	0.4387	0.1358	0.5970	0.059*	0.492 (5)
H11H	0.4805	0.1090	0.7075	0.059*	0.492 (5)
H11I	0.5101	0.0329	0.6390	0.059*	0.492 (5)
H1NB	0.669 (2)	0.0719 (16)	0.8648 (17)	0.043 (6)*	
H2NB	0.881 (2)	0.0033 (18)	0.9694 (18)	0.053 (7)*	
H2NA	0.384 (2)	0.029 (2)	0.9802 (19)	0.060 (8)*	
H1NA	0.204 (3)	0.1404 (18)	0.9184 (19)	0.052 (7)*	

### Atomic displacement parameters (Å^2^)

**Table:**

	*U*^11^	*U*^22^	*U*^33^	*U*^12^	*U*^13^	*U*^23^
O1A	0.0284 (7)	0.0292 (7)	0.0398 (9)	0.0114 (5)	0.0174 (7)	0.0106 (6)
N1A	0.0297 (9)	0.0254 (8)	0.0368 (10)	0.0094 (6)	0.0193 (8)	0.0101 (6)
N2A	0.0286 (8)	0.0247 (8)	0.0325 (9)	0.0087 (6)	0.0172 (7)	0.0083 (6)
C1A	0.0265 (9)	0.0235 (8)	0.0255 (10)	0.0036 (7)	0.0125 (8)	0.0027 (7)
C2A	0.0246 (9)	0.0250 (8)	0.0234 (10)	0.0043 (7)	0.0097 (7)	0.0020 (7)
C3A	0.0234 (9)	0.0223 (8)	0.0256 (10)	0.0022 (6)	0.0097 (7)	0.0024 (7)
C4A	0.0229 (9)	0.0237 (9)	0.0442 (13)	0.0030 (7)	0.0153 (9)	0.0036 (8)
C5A	0.0263 (10)	0.0321 (10)	0.0574 (15)	0.0007 (8)	0.0144 (10)	−0.0018 (10)
C6A	0.0270 (12)	0.0389 (13)	0.116 (3)	−0.0031 (9)	0.0309 (15)	−0.0020 (14)
C7A	0.0510 (16)	0.0381 (13)	0.141 (3)	0.0047 (11)	0.064 (2)	0.0232 (16)
C8A	0.0510 (15)	0.0508 (14)	0.100 (2)	0.0200 (11)	0.0524 (16)	0.0411 (14)
C9A	0.0293 (11)	0.0391 (11)	0.0591 (16)	0.0110 (8)	0.0255 (11)	0.0210 (10)
C10A	0.0316 (10)	0.0290 (9)	0.0372 (12)	0.0101 (7)	0.0203 (9)	0.0095 (8)
C11A	0.0548 (15)	0.0391 (12)	0.0558 (16)	0.0228 (10)	0.0375 (13)	0.0147 (10)
O1B	0.0221 (6)	0.0288 (6)	0.0264 (7)	0.0022 (5)	0.0111 (6)	0.0067 (5)
N1B	0.0201 (8)	0.0305 (8)	0.0294 (9)	0.0028 (6)	0.0076 (7)	0.0030 (6)
N2B	0.0189 (7)	0.0276 (7)	0.0221 (8)	0.0029 (5)	0.0087 (6)	0.0017 (6)
C1B	0.0321 (10)	0.0248 (9)	0.0266 (10)	0.0025 (7)	0.0049 (8)	0.0028 (7)
C2B	0.0249 (9)	0.0212 (8)	0.0217 (9)	0.0026 (6)	0.0129 (7)	0.0004 (6)
C3B	0.0307 (10)	0.0229 (8)	0.0213 (9)	0.0022 (7)	0.0104 (8)	0.0019 (7)
C4B	0.0410 (11)	0.0255 (9)	0.0186 (9)	−0.0031 (7)	0.0102 (8)	0.0008 (7)
C5B	0.0589 (14)	0.0258 (9)	0.0337 (12)	−0.0007 (9)	0.0294 (11)	0.0013 (8)
C6B	0.092 (2)	0.0353 (11)	0.0465 (15)	−0.0071 (11)	0.0504 (15)	−0.0031 (10)
C7B	0.104 (2)	0.0381 (12)	0.0357 (13)	−0.0155 (13)	0.0448 (15)	−0.0001 (10)
C8B	0.0787 (18)	0.0286 (10)	0.0245 (11)	−0.0093 (10)	0.0156 (12)	0.0046 (8)
C9B	0.0488 (13)	0.0281 (10)	0.0239 (10)	−0.0020 (8)	0.0081 (9)	0.0042 (7)
C10B	0.0427 (13)	0.0442 (13)	0.0400 (14)	0.0094 (10)	−0.0054 (11)	0.0093 (10)
C11B	0.032 (2)	0.053 (3)	0.033 (3)	0.0001 (18)	0.0032 (19)	0.0136 (19)
C11C	0.021 (2)	0.047 (3)	0.047 (3)	−0.0040 (17)	0.0109 (19)	0.014 (2)

### Geometric parameters (Å, °)

**Table:**

O1A—C2A	1.286 (2)	N1B—H1NB	1.00 (2)
N1A—C1A	1.350 (2)	N2B—C2B	1.356 (2)
N1A—N2A	1.372 (2)	N2B—H2NB	1.02 (3)
N1A—H1NA	0.98 (2)	C1B—C3B	1.386 (3)
N2A—C2A	1.353 (2)	C1B—C10B	1.497 (3)
N2A—H2NA	0.98 (3)	C2B—C3B	1.427 (2)
C1A—C3A	1.382 (2)	C3B—C4B	1.471 (3)
C1A—C10A	1.500 (2)	C4B—C5B	1.394 (3)
C2A—C3A	1.428 (2)	C4B—C9B	1.401 (3)
C3A—C4A	1.478 (2)	C5B—C6B	1.391 (3)
C4A—C5A	1.394 (3)	C5B—H5BA	0.9300
C4A—C9A	1.396 (3)	C6B—C7B	1.391 (3)
C5A—C6A	1.395 (3)	C6B—H6BA	0.9300
C5A—H5AA	0.9300	C7B—C8B	1.383 (4)
C6A—C7A	1.371 (4)	C7B—H7BA	0.9300
C6A—H6AA	0.9300	C8B—C9B	1.379 (3)
C7A—C8A	1.381 (4)	C8B—H8BA	0.9300
C7A—H7AA	0.9300	C9B—H9BA	0.9300
C8A—C9A	1.391 (3)	C10B—C11C	1.403 (3)
C8A—H8AA	0.9300	C10B—C11B	1.408 (3)
C9A—H9AA	0.9300	C10B—H10A	0.9700
C10A—C11A	1.520 (3)	C10B—H10B	0.9700
C10A—H10C	0.9700	C10B—H10E	0.9700
C10A—H10D	0.9700	C10B—H10F	0.9700
C11A—H11D	0.9600	C11B—H11A	0.9600
C11A—H11E	0.9600	C11B—H11B	0.9600
C11A—H11F	0.9600	C11B—H11C	0.9600
O1B—C2B	1.283 (2)	C11C—H11G	0.9600
N1B—C1B	1.352 (2)	C11C—H11H	0.9600
N1B—N2B	1.372 (2)	C11C—H11I	0.9600
			
C1A—N1A—N2A	108.56 (15)	N1B—N2B—H2NB	123.0 (14)
C1A—N1A—H1NA	131.6 (14)	N1B—C1B—C3B	109.01 (16)
N2A—N1A—H1NA	115.8 (14)	N1B—C1B—C10B	121.27 (19)
C2A—N2A—N1A	109.22 (15)	C3B—C1B—C10B	129.69 (19)
C2A—N2A—H2NA	128.0 (15)	O1B—C2B—N2B	122.08 (16)
N1A—N2A—H2NA	121.5 (15)	O1B—C2B—C3B	131.19 (16)
N1A—C1A—C3A	108.78 (15)	N2B—C2B—C3B	106.72 (16)
N1A—C1A—C10A	120.80 (16)	C1B—C3B—C2B	106.31 (16)
C3A—C1A—C10A	130.40 (16)	C1B—C3B—C4B	127.96 (17)
O1A—C2A—N2A	122.23 (16)	C2B—C3B—C4B	125.72 (17)
O1A—C2A—C3A	130.95 (17)	C5B—C4B—C9B	117.91 (18)
N2A—C2A—C3A	106.82 (15)	C5B—C4B—C3B	120.77 (16)
C1A—C3A—C2A	106.39 (15)	C9B—C4B—C3B	121.31 (18)
C1A—C3A—C4A	128.76 (15)	C6B—C5B—C4B	120.9 (2)
C2A—C3A—C4A	124.86 (16)	C6B—C5B—H5BA	119.5
C5A—C4A—C9A	118.53 (19)	C4B—C5B—H5BA	119.5
C5A—C4A—C3A	120.08 (18)	C5B—C6B—C7B	120.0 (2)
C9A—C4A—C3A	121.37 (17)	C5B—C6B—H6BA	120.0
C4A—C5A—C6A	120.4 (2)	C7B—C6B—H6BA	120.0
C4A—C5A—H5AA	119.8	C8B—C7B—C6B	119.7 (2)
C6A—C5A—H5AA	119.8	C8B—C7B—H7BA	120.1
C7A—C6A—C5A	120.3 (2)	C6B—C7B—H7BA	120.1
C7A—C6A—H6AA	119.8	C9B—C8B—C7B	120.1 (2)
C5A—C6A—H6AA	119.8	C9B—C8B—H8BA	119.9
C6A—C7A—C8A	120.2 (2)	C7B—C8B—H8BA	119.9
C6A—C7A—H7AA	119.9	C8B—C9B—C4B	121.3 (2)
C8A—C7A—H7AA	119.9	C8B—C9B—H9BA	119.3
C7A—C8A—C9A	120.0 (2)	C4B—C9B—H9BA	119.3
C7A—C8A—H8AA	120.0	C11C—C10B—C1B	120.6 (3)
C9A—C8A—H8AA	120.0	C11B—C10B—C1B	117.2 (2)
C8A—C9A—C4A	120.6 (2)	C11B—C10B—H10A	108.0
C8A—C9A—H9AA	119.7	C1B—C10B—H10A	108.0
C4A—C9A—H9AA	119.7	C11B—C10B—H10B	108.0
C1A—C10A—C11A	114.22 (16)	C1B—C10B—H10B	108.0
C1A—C10A—H10C	108.7	H10A—C10B—H10B	107.2
C11A—C10A—H10C	108.7	C11C—C10B—H10E	107.2
C1A—C10A—H10D	108.7	C1B—C10B—H10E	107.2
C11A—C10A—H10D	108.7	C11C—C10B—H10F	107.2
H10C—C10A—H10D	107.6	C1B—C10B—H10F	107.2
C10A—C11A—H11D	109.5	H10E—C10B—H10F	106.8
C10A—C11A—H11E	109.5	C10B—C11B—H11A	109.5
H11D—C11A—H11E	109.5	C10B—C11B—H11B	109.5
C10A—C11A—H11F	109.5	C10B—C11B—H11C	109.5
H11D—C11A—H11F	109.5	C10B—C11C—H11G	109.5
H11E—C11A—H11F	109.5	C10B—C11C—H11H	109.5
C1B—N1B—N2B	108.18 (15)	H11G—C11C—H11H	109.5
C1B—N1B—H1NB	128.5 (13)	C10B—C11C—H11I	109.5
N2B—N1B—H1NB	114.6 (14)	H11G—C11C—H11I	109.5
C2B—N2B—N1B	109.63 (15)	H11H—C11C—H11I	109.5
C2B—N2B—H2NB	126.9 (14)		
			
C1A—N1A—N2A—C2A	5.0 (2)	N2B—N1B—C1B—C3B	4.0 (2)
N2A—N1A—C1A—C3A	−3.5 (2)	N2B—N1B—C1B—C10B	−177.96 (18)
N2A—N1A—C1A—C10A	177.71 (17)	N1B—N2B—C2B—O1B	−179.06 (15)
N1A—N2A—C2A—O1A	175.93 (17)	N1B—N2B—C2B—C3B	1.65 (18)
N1A—N2A—C2A—C3A	−4.4 (2)	N1B—C1B—C3B—C2B	−2.9 (2)
N1A—C1A—C3A—C2A	0.8 (2)	C10B—C1B—C3B—C2B	179.2 (2)
C10A—C1A—C3A—C2A	179.43 (19)	N1B—C1B—C3B—C4B	177.89 (17)
N1A—C1A—C3A—C4A	−179.05 (19)	C10B—C1B—C3B—C4B	0.1 (3)
C10A—C1A—C3A—C4A	−0.4 (3)	O1B—C2B—C3B—C1B	−178.43 (18)
O1A—C2A—C3A—C1A	−178.1 (2)	N2B—C2B—C3B—C1B	0.77 (19)
N2A—C2A—C3A—C1A	2.2 (2)	O1B—C2B—C3B—C4B	0.8 (3)
O1A—C2A—C3A—C4A	1.7 (3)	N2B—C2B—C3B—C4B	179.96 (16)
N2A—C2A—C3A—C4A	−177.92 (18)	C1B—C3B—C4B—C5B	−134.8 (2)
C1A—C3A—C4A—C5A	138.1 (2)	C2B—C3B—C4B—C5B	46.2 (3)
C2A—C3A—C4A—C5A	−41.7 (3)	C1B—C3B—C4B—C9B	46.4 (3)
C1A—C3A—C4A—C9A	−43.5 (3)	C2B—C3B—C4B—C9B	−132.6 (2)
C2A—C3A—C4A—C9A	136.7 (2)	C9B—C4B—C5B—C6B	−1.6 (3)
C9A—C4A—C5A—C6A	0.2 (3)	C3B—C4B—C5B—C6B	179.5 (2)
C3A—C4A—C5A—C6A	178.6 (2)	C4B—C5B—C6B—C7B	0.6 (4)
C4A—C5A—C6A—C7A	0.4 (4)	C5B—C6B—C7B—C8B	0.4 (4)
C5A—C6A—C7A—C8A	−0.6 (4)	C6B—C7B—C8B—C9B	−0.2 (4)
C6A—C7A—C8A—C9A	0.2 (4)	C7B—C8B—C9B—C4B	−0.8 (3)
C7A—C8A—C9A—C4A	0.4 (4)	C5B—C4B—C9B—C8B	1.8 (3)
C5A—C4A—C9A—C8A	−0.6 (3)	C3B—C4B—C9B—C8B	−179.4 (2)
C3A—C4A—C9A—C8A	−179.0 (2)	N1B—C1B—C10B—C11C	−22.8 (4)
N1A—C1A—C10A—C11A	33.3 (3)	C3B—C1B—C10B—C11C	154.8 (3)
C3A—C1A—C10A—C11A	−145.2 (2)	N1B—C1B—C10B—C11B	−96.9 (3)
C1B—N1B—N2B—C2B	−3.53 (19)	C3B—C1B—C10B—C11B	80.7 (3)

### Hydrogen-bond geometry (Å, °)

**Table:**

Cg1 and Cg2 are the centroids of the C4B–C9B and C4A–C9A rings, respectively.

**Table:**

*D*—H···*A*	*D*—H	H···*A*	*D*···*A*	*D*—H···*A*
N1B—H1NB···O1A	1.00 (2)	1.73 (2)	2.700 (2)	161 (2)
N2B—H2NB···O1B^i^	1.02 (2)	1.72 (2)	2.738 (2)	176 (2)
N2A—H2NA···O1A^ii^	0.98 (3)	1.74 (3)	2.704 (2)	171 (2)
N1A—H1NA···O1B^iii^	0.98 (3)	1.74 (3)	2.691 (2)	162 (2)
C8A—H8AA···O1A^iv^	0.93	2.47	3.370 (3)	163
C10A—H10C···Cg1^iii^	0.97	2.61	3.464 (2)	147
C10B—H10E···Cg2	0.97	2.71	3.524 (3)	142

Symmetry codes: (i) −*x*+2, −*y*, −*z*+2; (ii) −*x*+1, −*y*, −*z*+2; (iii) *x*−1, *y*, *z*; (iv) −*x*+1, *y*+1/2, −*z*+3/2.
